# G-1 exerts neuroprotective effects through G protein-coupled estrogen receptor 1 following spinal cord injury in mice

**DOI:** 10.1042/BSR20160134

**Published:** 2016-08-31

**Authors:** Qiang Cheng, Jia Meng, Xin-shang Wang, Wen-bo Kang, Zhen Tian, Kun Zhang, Gang Liu, Jian-ning Zhao

**Affiliations:** * Department of Orthopedics, Jinling Hospital, Clinical School of Nanjing, Second Military Medical University, Nanjing 210002, China; † Department of Pharmacology, School of Pharmacy, Fourth Military Medical University, Xi'an 710032, China

**Keywords:** apoptosis, GPR30, motor functional recovery, neuroprotection

## Abstract

Spinal cord injury (SCI) always occurs accidently and leads to motor dysfunction because of biochemical and pathological events. Estrogen has been shown to be neuroprotective against SCI through estrogen receptors (ERs), but the underlying mechanisms have not been fully elucidated. In the present study, we investigated the role of a newly found membrane ER, G protein-coupled estrogen receptor 1 (GPR30 or GPER1), and discussed the feasibility of a GPR30 agonist as an estrogen replacement. Forty adult female C57BL/6J mice (10–12 weeks old) were divided randomly into vehicle, G-1, E2, G-1 + G-15 and E2 + G-15 groups. All mice were subjected to SCI using a crushing injury approach. The specific GPR30 agonist, G-1, mimicked the effects of E2 treatment by preventing SCI-induced apoptotic cell death and enhancing motor functional recovery after injury. GPR30 activation regulated phosphatidylinositol 3-kinase (PI3K)/Akt and MAPK/extracellular signal-regulated kinase (ERK) signalling pathways, increased GPR30 and anti-apoptosis proteins Bcl-2 and brain derived neurotrophic factor (BDNF), but decreased the pro-apoptosis factor Bax and cleaved caspase-3. However, the neuroprotective effects of G-1 and E2 were blocked by the specific GPR30 antagonist, G-15. Thus, GPR30 rather than classic ERs is required to induce estrogenic neuroprotective effects. Given that estrogen replacement therapy may cause unexpected side effects, especially on the reproductive system, GPR30 agonists may represent a potential therapeutic approach for treating SCI.

## INTRODUCTION

Numerous studies have verified that estrogen exhibits clear neuroprotective effects following stroke and cerebral ischemia [1–3], and estrogen replacement therapy (ERT) is a well-established method of managing climacteric symptoms in women after menopause [4]. However, several clinical trials on ERT found that estrogen increases the risk of coronary heart diseases and breast cancers [5,6], which greatly restricts its clinical applications. Estrogen exerts effects by binding to classic estrogen receptors α and β (ERα and ERβ) and a putative membrane estrogen receptor, GPR30 [7]. Most studies have demonstrated that classic ERs play an indispensable role in estrogenic neuroprotection [8]. However, GPR30 knockout attenuates estrogen-induced neuroprotection and activation of a rapid kinase signal pathway in global cerebral ischemia [9]. Similar to estrogen, the specific GPR30 agonist, G-1, has been reported to significantly increase the amplitude of excitatory postsynaptic currents [10,11]. Furthermore, G-1 exerts neuroprotective effects against NMDA-induced oxidative toxicity *in vitro* [12] and inhibits osteoporosis in ovariectomized (OVX) rats [13].

Spinal cord injury (SCI) is severe and common accident in daily life. The incidence of SCI is approximately 40 cases per million population with 12000 new cases every year, according to the National Spinal Cord Injury database (2011). If SCI was not treated properly, secondary damage consisting of complex pathological events might lead to paralysis and motor dysfunction [14]. Due to the complexity of the central nervous system (CNS), the underlying mechanisms of SCI remain ill-defined, and no effective drug has been identified so far.

It has been reported that classic ERs are mainly distributed in the superficial layer of the dorsal horn of the spinal cord [15,16]. In contrast, GPR30 is mainly distributed in the motoneurons of the ventral horn and white matter of the spinal cord [17], both of which are directly associated with motor function. The tissue distribution characteristics of GPR30 led to the hypothesis that GPR30 might be a candidate for promoting recovery of SCI-induced motor dysfunction. The present study aimed to explore the effects of GPR30 activation on SCI and its underlying molecular mechanisms, which may provide a new strategy for treatment of SCI.

## MATERIALS AND METHODS

### Materials

**Figure 1:**
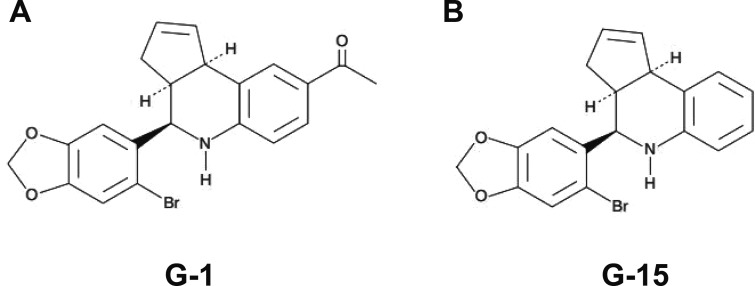
(**A**) Chemical structures of G-1. (**B**) Chemical structures of G-15. G-1 and G-15 competitively bind with GPR30 and display low affinity with the classical ERs.

G-1, G-15 and 17β-oestradiol (E2) were obtained from Cayman Chemical and dissolved in olive oil (Bellina, Baena, Spain) as previously described [18]. G-1 (Figure 1A) is a nonsteroidal, high-affinity, selective agonist of GPR30 (*K_i_*=11 nM). G-15 (Figure 1B) is a non-steroidal antagonist of GPR30 (*K_i_*=20 nM). They display low affinity cross-reactivity with the classical ERs [19]. Other chemicals and reagents were commercially available and of standard biochemical quality.

### Animals

Forty adult female C57BL/6J mice (22–25 g) were housed at 25±2°C in a room with a 12-h light/dark cycle and free access to food and water for 1 week. Then they were randomly divided into four groups: (1) vehicle group (olive oil, *n*=8), (2) G-1 group (G-1, 1 μg/mice daily, *n*=8), (3) E2 group (estrogen, 1 μg/mice daily, *n*=8), (4) E2 + G-15 group (E2, 1 μg/mice daily; G-15, 5 μg/mice daily, *n*=8), (5) G-1 + G-15 group (G-1, 1 μg/mice daily; G-15, 5 μg/mice daily, *n*=8). The doses of G-1, G-15 and E2 used here were based on a previous study [20]. Mice were anaesthetized with chloral hydrate (5%, 0.2 ml/30 g body weight, intraperitoneally), and a modified dorsal laminectomy operation was performed at T7–T9 to expose the spinal cord as described by Marques [21]. Then the spinal cord was clipped for 20 s using a modified micro-ophthalmic forcep (53327T, 66 Vision-Tech), which was modified with an attached cushion to maintain a distance of 0.2 mm between the blades. This procedure led to a moderate SCI as shown by previous research [22,23]. Mouse subjected to surgery was administered subcutaneously 1 ml of saline to replace blood volume lost during surgery and then kept on a 37°C heating pad until it regained consciousness. Urine was manually expressed twice daily until the bladder emptying reflex recovered, and mice were treated with ampicillin (10 mg/kg) once daily for 3 days to prevent infection. E2, G-1 and G-15 were diluted in olive oil and intraperitoneally injected for 14 days. The vehicle group received an equal volume of olive oil. All procedures were conducted in accordance with the Animal Ethics Committee of the Fourth Military Medical University.

### Basso mouse scale

Basso mouse scale (BMS) is a widely used test that evaluates whole motor function from complete paralysis to normal locomotion [24]. As the complement of the BMS score, the BMS subscore reflects specific changes such as stepping frequency, coordination, paw position, trunk stability and tail position. The BMS was assessed 1 day and 14 days after SCI in a box (150 cm long, 120 cm wide and 30 cm high).

### Inclined plane test

The inclined plane test (IPT) is used to assess gross motor function in animal models with severe injury, such as SCI [25,26]. The mouse is placed on an inclined board to assess its ability to maintain position for at least 5 s without falling. The board is raised in 5° increments during the process, and the maximum angle at which the mouse can maintain position is recorded. Mice were tested before surgery and 14 days after SCI. Mice were allowed to move freely for 5 min before testing, and the behavioural tests were assessed blindly by two trained investigators.

### Histological processing

Four randomly selected animals from each group were killed by chloral hydrate overdose (5%, 0.5 ml/30 g) 14 days post-injury for staining and immunohistochemistry. Mice were perfused through a cannula inserted into the ascending aorta with 20 mL warm normal saline followed by 20 mL paraformaldehyde in 0.1 M phosphate buffer (4°C). After perfusion, 0.5 cm-long spinal cord segments centred within the injured site were collected and postfixed in 4% paraformaldehyde. The spinal cords underwent manual processing through alcohols, chloroform, and paraffin wax for paraffin embedding. The cross sections (8–10 μm thick) were used for the following morphological staining procedures. To characterize the tissue damage after SCI, the sections were stained with haematoxylin and eosin (H&E), Luxol fast blue-cresyl violet (LFB), and by caspase 3 immunohistochemistry.

### HE staining

The cross sections were rinsed in distilled water and stained in haematoxylin solution for 5 min. After washing with running tap water for 5 min, they were differentiated in 1% acid alcohol for 30 s and washed again with tap water for 1 min. Then the sections were immersed in eosin for 30 s and dehydrated through 70%, 80%, 90% and 100% alcohol for 2 min each.

### LFB staining

The cross sections were rinsed in distilled water, stained in 1% LFB solution (Sigma–Aldrich) for 2 h at 60°C, and then differentiated with 0.05% lithium carbonate solution to distinguish white and grey matter of the spinal cord. Subsequently, the sections were immersed in 1% cresyl violet solution for 10 min and quickly dehydrated with 70% and 95% alcohol until myelinated tracts of the spinal columns were stained blue [27]. Three sections per mice were chosen for assessing the myelinated area of spinal white matter.

### Immunohistochemistry

Cross sections centred within the injured part of the spinal cord segments were incubated with 3% H_2_O_2_ to eliminate endogenous peroxidase activity and then washed several times in phosphate-buffered saline. After incubating in 0.15% Triton X-100 at room temperature and blocking with 1% goat serum albumin in modified D-PBS Tween-20 for 1 h, the sections were incubated overnight with rabbit anti-caspase 3 antibody (1:200, Millipore). Next, sections were incubated with horseradish peroxidase-conjugated secondary antibody for 2 h. Diaminobenzidine served as the substrate for peroxidase.

### Image analysis

The cross-sectional cavity area was measured to determine the extent of damage, and the cross-sectional area stained with Luxol was also calculated to evaluate damage of myelinated tracts [28]. The caspase-3 positive cells in cross sections were calculated to evaluate the extent of cell apoptosis in the spinal cord. These cross sections were examined at 40× and 200× magnification using brightfield microscopy (Olympus BX61, Tokyo, Japan) with an Image-Pro Plus image analysis system (Version 6.0, Cybernetics). Cavity area/total cross-sectional area, myelinated white matter area/total cross-sectional area and counting of caspase-3 positive cells were well-accepted quantitative techniques to assess lesion size and were used to evaluate biological differences between different treatment groups. The results of vehicle group were taken as 1, and the rest results of other treatment groups were expressed as percentage to the vehicle group.

### Western blotting

The remaining 20 mice were killed by cervical dislocation, and 0.5 cm-long fresh spinal cord segments were collected around the site of injury and stored at -80°C for western blot analysis. Equal amounts of protein (50 μg) were separated and transferred on to PVDF membranes (Invitrogen). The membranes were probed with antibodies for brain derived neurotrophic factor (BDNF) (1:1000, Sigma–Aldrich), Bcl-2 (1:1000, Abcam), Bax (1:1000, Abcam), p-Akt (1:1000, Abcam), p-ERK (1:1000, Abcam), cleaved caspase-3 (1:200, Millipore) and β-actin (1:10000, Abcam). The membranes were then incubated with horseradish peroxidase-conjugated secondary antibodies (anti-rabbit/anti-mice IgG for the primary antibodies). Densitometric analysis of the western blots was conducted using a ChemiDoc XRS (Bio-Rad Laboratories), and signals were quantified using Quantity One version 4.1.0 (Bio-Rad Laboratories) according to the manufacturer's instructions. For data quantification of each blot, band density was calculated relative to β-actin. In addition, the ratio of the vehicle group was set as 1, and the band densities of other treatment groups were expressed as percentages of the vehicle group.

### Data analysis

Data were analysed using SPSS V.13.0 (SPSS, Chicago, IL, USA), and bars represent mean±S.E.M. Data that passed the homogeneity test were analysed using one-way ANOVA followed by the least significant difference (LSD) test. Otherwise, data were analysed using one-way ANOVA with Dunnett's T3 test for comparisons. In all cases, *P*< 0.05 was considered statistically significant.

## RESULTS

### GPR30 activation promoted mice hindlimb motor function after SCI

**Figure 2:**
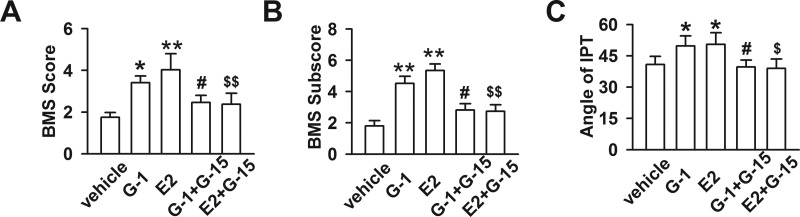
Effects of GPR30 on BMS scores and IPT 14 days after SCI (**A**) BMS scores in the G-1 and E2 groups were significantly higher than the vehicle group (*F*_(4, 35)_=5.071, *P* < 0.05). (**B**) BMS subscores paralleled the BMS scores (*F*_(4, 35)_=6.835, *P* < 0.05). (**C**) The maximum angle maintained on the inclined plane declined sharply 14 days after SCI but was restored by E2 or G-1 (*F*_(4, 35)_=3.429, *P* < 0.05). *n*=8, *P < 0.05, **P < 0.01 compared with vehicle group; ^#^*P* < 0.05, ^##^*P* < 0.01 compared with G-1 group; ^$^*P* < 0.05, ^$$^*P* < 0.01 compared with E2 group.

Motor functions of mice hindlimbs were evaluated via the BMS and IPT as described above. Before SCI, BMS scores, BMS subscores and IPT angles were tested to ensure that all the mice had similar initial locomotor activities. At 1 day after acute SCI, the BMS scores of all groups decreased sharply to 0, and their hindlimbs appeared paralysed, suggesting that the SCI animal model was well established. Over time, mice motor function gradually recovered. The results at 14 days post-injury showed that the BMS scores were significantly higher in the G-1 and E2 groups compared with the other groups (*P* < 0.05; Figure 2A). There was no significant difference between the G-1 and E2 groups, as well as the other three groups. BMS subscores were similar to the BMS scores (Figure 2B). The results of IPT (Figure 2C) expressed the similar trend as BMS scores.

### GPR30 activation improved tissue repair after SCI

**Figure 3:**
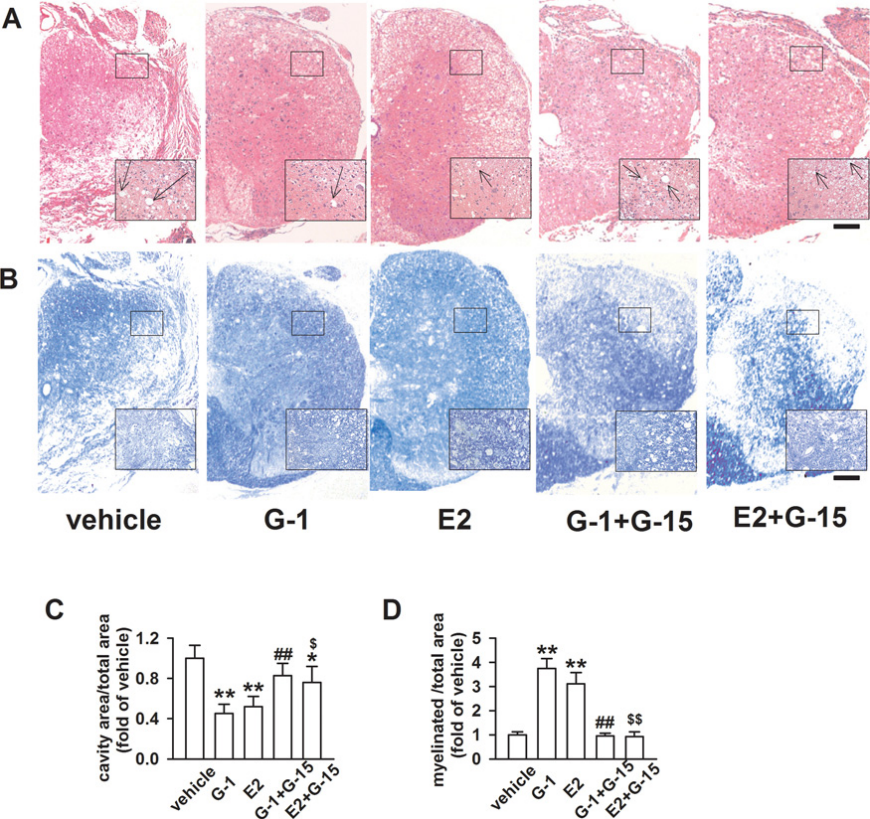
Effects of GPR30 on morphological degenerations of the spinal cord 14 days after SCI (**A**) HE staining images of the spinal cord ventral horn (40× and 200× magnification, scale bars=100 μm). Arrows, zones of cavitation. (**B**) LFB staining images of the spinal cord ventral horn (40× and 200× magnification, scale bars=100 μm). (**C**) G-1 and E2 treatment decreased the ratio of cavity area/total area obviously, and the E2 + G-15 group also showed slightly decline (*F*_(4, 15)_=13.063, *P* < 0.01). (**D**) G-1 and E2 increased the ratio of myelinated area/total area, but G-15 blocked their effects (*F*_(4, 15)_=17.356, *P* < 0.01). *n*=4, *P < 0.05, **P < 0.01 compared with vehicle group; ^#^*P* < 0.05, ^##^*P* < 0.01 compared with G-1 group; ^$^*P* < 0.05, ^$$^*P* < 0.01 compared with E2 group.

Secondary biochemical damage following SCI led to morphological degenerations, which were the basis of motor dysfunction. We found extensive swelling at the crush site following moderate SCI. HE staining of cross sections showed numerous inflammatory and shrunken cells, and cavitations were found in local high definition images (Figure 3A). To quantify the extent of lesions in the spinal cord, the ratio of cavity area/total area in HE staining was determined. The ratio in the vehicle, G-1 + G-15 and E2 + G-15 groups increased significantly. In contrast, the ratio in the G-1 and E2 groups decreased compared with the vehicle group (*P* < 0.05; Figure 3C), demonstrating that G-1 and E2 could inhibit cavitation in the spinal cord. Myelinated areas of cross sections stained dark blue, and other area was white in LFB staining. The images of LFB staining showed that the vehicle, G-1 + G-15 and E2 + G-15 groups had less myelin (Figure 3B). The ratio of myelinated area/total area was used to quantify the extent of white matter lesions. We found ratios for the G-1 and E2 groups were higher compared with the vehicle group (*P* < 0.05, Figure 3D).

### GPR30 activation alleviated cell apoptosis after SCI

**Figure 4:**
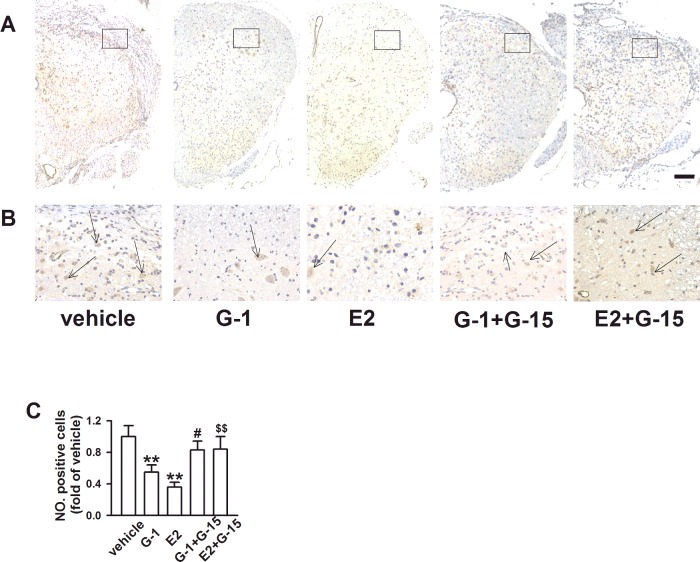
Effects of GPR30 activation on cell apoptosis in the spinal cord 14 days after SCI (**A**) Among the five groups, the number of caspase-3 positive cells was highest in the vehicle group and lowest in the E2 group (40× magnification, scale bars=100 μm). (**B**) Higher magnification images of the spinal cord ventral horn from different groups (200× magnification). Arrows, typical apoptotic cells. (**C**) E2 and G-1 groups expressed fewer caspase-3-positive cells compared with other groups (*F*_(4, 15)_=15.148, *P* < 0.01). *n*=4, *P < 0.05, **P < 0.01 compared with vehicle group; ^#^*P* < 0.05, ^##^*P* < 0.01 compared with G-1 group; ^$^*P* < 0.05, ^$$^*P* < 0.01 compared with E2 group.

Caspase-3 immunohistochemical staining was used to assess the effects of GPR30 activation on cell apoptosis in the spinal cord after SCI (Figure 4A), and represent images of high definition for caspase-3 positive cells in different groups were showed in Figure 4(B). The number of caspase-3-positive cells in the cross sections was counted, and G-1 and E2 groups had fewer positive cells compared with the other groups (*P* < 0.05; Figure 4C), and there was no difference between the two groups (*P* >  0.05; Figure 4C), but the rest three groups showed no difference (*P* >  0.05; Figure 4C). These results were consistent with those of H&E and LFB stainings.

### GPR30 activation increased expression of GPR30 and G-15 blocked it

**Figure 5:**
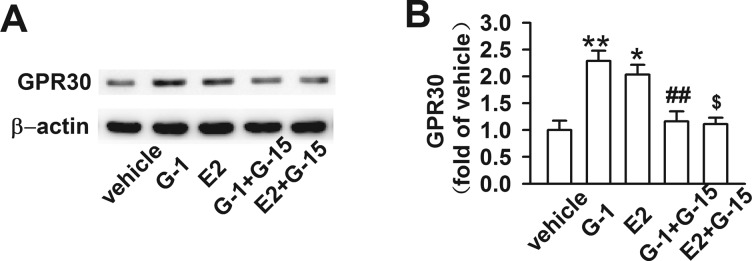
Expression of GPR30 following treatment with G-1, E2 or/and G-15 (**A**) Representative images of western blot analysis for GPR30. (**B**) Expression of GPR30 in the G-1 and E2 groups remained at higher levels compared with that of the vehicle and E2 + G-15 groups (*F*_(4, 15)_=8.481, *P* < 0.05). The increase in GPR30 in G-1 and E2 groups might represent a compensatory reaction for exogenous stimulations on GPR30. *n*=4, *P < 0.05, **P < 0.01 compared with vehicle group; ^#^*P* < 0.05, ^##^*P* < 0.01 compared with G-1 group; ^$^*P* < 0.05, ^$$^*P* < 0.01 compared with E2 group.

Represent images of western blot analysis for GPR30 in different groups were showed in Figure 5(A). Using western blot analysis, we found that G-1 or E2 treatment clearly increased the expression of GPR30 compared with the vehicle group, possibly acting as a compensatory reaction for high level of G-1 or E2 (*P* < 0.05; Figure 5B). In contrast, the GPR30 expression in G-1 + G-15 or E2 + G-15 group showed no significant difference with the vehicle group (*P* >  0.05; Figure 5B). We hypothesized that the activation of GPR30 might be counteracted by G-15, because G-15 was five times of G-1 or E2 and had high affinity with GPR30 as described above.

### GPR30 activation regulated associated proteins through PI3K/Akt and MAPK/ERK signalling pathways

**Figure 6:**
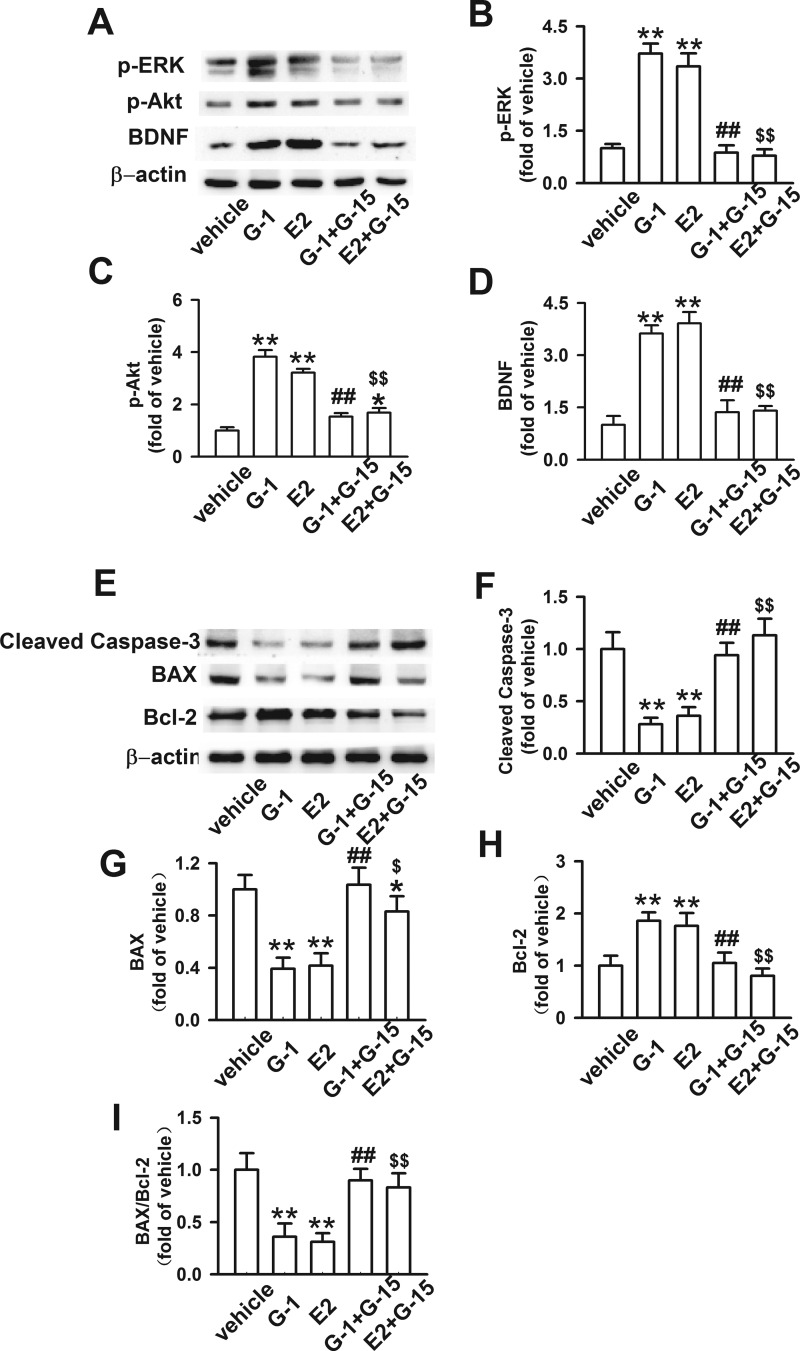
Effects of GPR30 on associated proteins via the PI3K/Akt and MAPK/ERK signalling pathways (**A**) Representative western blot image showing p-Akt, p-ERK and BDNF. (**B**) Levels of p-ERK increased after treatment with G-1 or E2 (*F*_(4, 15)_=21.310, *P* < 0.01). (**C**) Expression of p-Akt was highest in the G-1 group (*F*_(4, 15)_=15.093, *P* < 0.01). (**D**) The trend in BDNF levels was similar to that of p-Akt and p-ERK (*F*_(4, 15)_=17.528, *P* < 0.01). (**E**) Representative western blot images showing the levels of cleaved caspase-3, Bax and Bcl-2. (**F**) Levels of cleaved caspase-3 decreased in G-1 and E2 groups after SCI (*F*_(4, 15)_=14.528, *P* < 0.01). (**G**) Levels of BAX in the vehicle and G-1 + G-15 groups were much higher compared with the vehicle groups (*F*_(4, 15)_=11.273, *P* < 0.01). (**H**) Bcl-2 in G-1 and E2 groups showed significant differences compared with the vehicle groups (*F*_(4, 15)_=8.492, *P* < 0.05). (**I**) The ratio of Bax/Bcl-2 was consistent with the trend of BAX. *n*=4, *P < 0.05, **P < 0.01 compared with vehicle group; ^#^*P* < 0.05, ^##^*P* < 0.01 compared with G-1 group; ^$^*P* < 0.05, ^$$^*P* < 0.01 compared with E2 group.

Phosphatidylinositol 3-kinase (PI3K)/Akt and MAPK/extracellular signal-regulated kinase (ERK) signal pathways were cooperated in E2-induced neuroprotective effects by promoting synthesis of BDNF (Figure 6A). Expressions of p-Akt and p-ERK in the E2 and G-1 groups were higher than those in the G-1 + G-15 and E2 + G-15 group (*P* < 0.01; Figures 6B and 6C), and the trend of BDNF was similar to that of p-Akt and p-ERK (Figure 6D). Cleaved caspase-3 was the active form of caspase-3, reflecting the cell apoptosis condition in the spinal cord. Bcl-2 and Bax were considered downstream cascades of PI3K/Akt and MAPK/ERK signalling pathways (Figure 6E). We found that the expression of cleaved caspase-3 in G-1 and E2 groups were lower than the other three groups (*P* < 0.05; Figure 6F). A similar situation happened in Bax, but the BAX level in E2 + G-15 group showed a difference compared with the vehicle group (*P* < 0.05; Figure 6G). In contrast, Bcl-2 levels were elevated in the G-1 and E2 groups (Figure 6H). The change was more evident in the ratio of Bax/Bcl-2: the ratios for the G-1 and E2 groups were significantly lower compared with the rest groups (*P* < 0.05; Figure 6I).

## DISCUSSION

Estrogen has been widely reported to be neuroprotective against neurodegenerative diseases [29,30], it is identified that estrogen exerts effects by activating ERs, but the underlying mechanisms are not fully understood. Classic ERs are expressed in the superficial laminae of the dorsal horn of the spinal cord, which is mainly involved with sensory and nociceptive modulation [31,32]. GPR30 is mainly expressed in neurons and white matter of the ventral horn of the spinal cord, both of which are closely related to motor function [17]. Evidence from human research has shown that there is little ERs immunostaining in the ventral horn of spinal cord [33]. The special distribution pattern of GPR30 might be the basis of GPR30-mediated estrogenic neuroprotection against SCI. The present study provided strong evidence for the role of GPR30 in SCI. The specific GPR30 agonist, G-1, had a protective effect similar to E2 in reducing SCI-induced apoptosis and promoting recovery of motor function. Application of the specific GPR30 antagonist, G-15, together with G-1 or E2, could nearly counteract the effects induced by G-1 or E2 alone. Although E2+G-15 showed some improvements in some indexes here, the process might be induced by classic ERs, which had little relationship with mice motor function because of the characteristic distributions of classic ERs [6,15]. Therefore, our data, taken together with other studies, support the hypothesis that GPR30 activation might mediate estrogenic neuroprotection against SCI and play a critical role in recovery of mice hindlimb motor function.

Previous results have shown that E2 is effective in neuroprotection [34,35]. However, few studies have examined the neuroprotective role of GPR30 in E2-mediated neuroprotection, and the interplay of classic ERs and GPR30 was not elucidated. Some studies have indicated that ERα might be more important in injury-induced E2-mediated protection [36], whereas ERβ may only play a role in basal neuroprotection [37]. However, we found that G-1 with similar concentration of E2 could effectively protect spinal motoneurons in SCI-treated mice. Our previous study verified that this physiological concentration of E2 could significantly inhibit osteoporosis induced by OVX in rats [38], and alleviate collagen-induced arthritis and immune-associated bone loss [20]. In this experiment, activation of GPR30 with G-1 could reproduce neuroprotective effects of E2, but we could not exclude the involvement of classic ERs in the development of neuroprotection, because there were endogenous estrogens existing in mice. To differentiate the role of GPR30 or ERs in the development of SCI, G-15, exhibiting a higher relative binding affinity than G-1 [39], was used to block activation of GPR30. We found G-15 depressed the neuroprotective effects in both G-1 and E2-treated mice, suggesting the protective effects exerted by G-1 were not influenced by endogenous estrogens of female mice. An *in vitro* study of cortical neurons has reported that protection against oxidative insult by G-1 is not blocked by ICI 182,780, an ER antagonist, suggesting the specific effect of GPR30 is independent of ERs [40], which is partly consistent with our results. Furthermore, GPR30 knockout mice showed a lack of protection of dopaminergic neurons by E2 [41]. In the present study, the increased expression of GPR30 in G-1 and E2 groups may be a compensatory reaction to E2 or G-1, but this process could be blocked by five times of G-15 competitively. Thus, we conclude that GPR30 is required to induce an estrogenic neuroprotective effect, and E2 exerts neuroprotection via GPR30 rather than classic ERs in the procedure.

Although E2 and G-1 could exert protective effects on motoneurons, but the role of GPR30 in mediating rapid estrogenic signalling has received little attention. Investigation of the underlying signalling pathways reveal common mechanisms but also some different actions. Previous studies found that phosphatidylinositol 3-kinase (PI3K) and mitogen-activated protein kinase (MAPK) signalling pathways are implicated in neuroprotective effects of E2 in 1-methyl-4-phenyl-1,2,3,6-tetrahydropyridine (MPTP)-treated mice and 6-hydroxydopamine-lesioned rats [42,43]. In addition, the increases in p-Akt, p-ERK and BDNF levels were blocked by G-15 in hippocampal neurons [9]. BDNF plays an important role in supporting survival of neurons, enhancing remyelination of injured axons, and decreasing necrosis in the CNS [44]. Bax and Bcl-2 are the downstream proteins of the PI3K/Akt and MAPK/ERK signal pathways [45]. In the present study, GPR30 activation promoted Akt and ERK phosphorylation levels, increased Bcl-2 and BDNF, and decreased the levels of cleaved caspase-3, BAX and the ratio of BAX/Bcl-2 in the G-1 and E2 groups. However, inhibition of GPR30 affected their expression levels in the E2 + G-15 and G-1+G-15 groups, suggesting that GPR30-mediated preservation of neurons in the spinal cord.

Several studies have identified neuroprotective effects of estrogen, but clinical trials on ERT were interrupted prematurely due to increased risks of coronary heart disease and breast cancer [46]. The intraperitoneal injection of the GPR30 agonist G1 for 14 days induces neuroprotective effects similar with the same dose of E2. We infer that E2 exert neuroprotection probably by the nonclassic ER, GPR30, because the effects of E2 could be blocked by G-15. Thus, manipulations that target GPR30 may be selective and effective strategies for SCI. The features of GPR30 endow it with more clinical significance as a novel alternative for treating both males and females and avoid peripheral risks associated with ERT. However, the safety of long-term application of GPR30 agonist needs further validation in the future.

## Abbreviations

BDNF: brain derived neurotrophic factor
BMS: Basso mouse scale
CNS: central nervous system
ER: estrogen receptor
ERK: extracellular signal-regulated kinase
G-1: rel-1-[4-(6-bromo-1,3-benzodioxol-5-yl)-3aR, 4S, 5, 9bS-tetrahydro-3H-cyclopenta[c]quinolin-8-yl]-ethanone
G-15: (3aS, 4R, 9bR)-4-(6-bromo-1,3-benzodioxol-5-yl)-3a, 4, 5, 9b-tetrahydro-3H-cyclopenta[c]quinoline
GPR30/GPER1: G protein-coupled estrogen receptor 1
H&E: haematoxylin and eosin
IPT: inclined plane test
LFB: Luxol fast blue
MAPK: mitogen-activated protein kinase
OVX: ovariectomy
PI3K: phosphatidylinositol 3-kinase
SCI: spinal cord injury
